# Supplementary material for: Driving through stop signs: predicting stop codon reassignment improves functional annotation of bacteriophages
Source: ISME Commun. 2024 Jun 19;4(1):ycae079. doi: 10.1093/ismeco/ycae079 (PMC11210395; doi:10.1093/ismeco/ycae079)
Supplement: Cook_Supplementary_Materials_ycae079 [file cook_supplementary_materials_ycae079.docx]

**Supplementary Methods**

**Datasets**

A multifasta file of phage genomes was downloaded from INPHARED (<https://github.com/RyanCook94/inphared>; September 2023)^10^. Stop codon reassignment of INPHARED genomes was predicted using Prodigal-gv v2.11.0 (<https://github.com/apcamargo/prodigal-gv>), a fork of Prodigal written to improve viral gene calling^13^. Those predicted to use translation table 4 or 15 were retained for downstream analysis.

The Unified Human Gut Virome Catalog (UHGV) was filtered for high quality and complete vOTUs deemed to be a “high confidence” virus and predicted to use either translation table 4 or 15 (<https://github.com/snayfach/UHGV>). Stop codon reassignment had already been predicted for UHGV vOTUs using Prodigal-gv and is available in the UHGV metadata.

**Prodigal-gv**

Stop codon reassignment and open reading frame (ORF) prediction was performed using Prodigal-gv v2.11.0 (<https://github.com/apcamargo/prodigal-gv>), a fork of Prodigal^13,14^. Prodigal-gv builds on Prodigal's metagenome mode by scanning each sequence through multiple pre-trained gene models and selecting the model that yields the highest score, ensuring the most accurate gene prediction according to Prodigal's scoring system. Unlike standard Prodigal, which utilizes models based on translation tables 4 and 11, prodigal-gv includes models that use translation table 15. These additional models are trained on phage genomes with stop codon reassignments, manually selected from the UHGV database. By utilizing models with translation tables that match the input genomes, prodigal-gv avoids low gene scores resulting from the prediction of short genes with premature stop codons when using an incorrect translation table.

**Prokka**

A fork of Prokka v1.14.5^17^ was written that incorporates an initial stage of ORF prediction using Prodigal-gv v2.11.0 (<https://github.com/apcamargo/prodigal-gv>)^13^. A first gene calling step is used to infer the genetic code most likely adopted by the genome, then the predicted genetic code is used to perform the translation FASTX::Seq, which we updated to accept code 15 ([metacpan.org/pod/FASTX::Seq](https://metacpan.org/pod/FASTX::Seq))^22^. The code for this is available at ([github.com/telatin/metaprokka](https://github.com/telatin/metaprokka)). We included publicly available HMMs of the PHROGs database in our Prokka-gv annotations (<http://s3.climb.ac.uk/ADM_share/all_phrogs.hmm.gz>)^23^. The fork is installable from Bioconda as ‘metaprokka’.

**Pharokka**

Pharokka v1.5.0^18^ was updated to include support for pyrodigal-gv implementing pyrodigal-gv as a gene predictor. This is specified by using ‘-g prodigal-gv’ when running Pharokka. The updated code is available on GitHub (<https://github.com/gbouras13/pharokka>). Pharokka uses tRNAscan-SE for predicting tRNAs^20^.

**Statistical Analyses and Data Visualisation**

To test for significance in differences of results, a paired sample T test was performed in R v4.2.2^24^ and P-values were adjusted using the Benjamini-Hochberg procedure^25^. Figure 1 was produced using ggplot2 v3.4.2^26^.

**Supplementary Results**

**Prokka-gv Annotations**

For Prokka-gv, the largest differences were observed for sequences predicted to use translation table 15, for which Prokka-gv increased the median gene length (median of per genome medians) from 276 to 396 bp for UHGV sequences (43.5% increase), and from 309 to 483 bp for INPHARED sequences (56.3% increase). This was also reflected in an increase of median coding capacity from 66.6% to 86.7% for UHGV, and from 69.2% to 87.3% for INPHARED. As it is commonly used as a phylogenetic marker for bacteriophages, we investigated how commonly the major capsid protein (MCP) could be identified with and without predicted stop codon reassignment^15^. For sequences predicted to use translation table 15, the MCP could be identified on 382/715 (53.4%) sequences with Prokka and this was marginally increased to 386/715 (53.9%) with Prokka-gv.

When investigating the sequences for which translation table 4 was predicted, a substantial increase was also observed for UHGV sequences, with Prokka-gv increasing median median gene length from 319 to 460 bp (44.2%), resulting in an increase of coding capacity from 78.4% to 91.4%. However, the same was not observed for INPHARED sequences predicted to use translation table 4. These sequences observed a modest increase in median median gene length from 573 to 584 bp (1.8%) for Prokka-gv. Median coding capacity was not increased with Prokka and Prokka-gv both obtaining 86.2%.

**Supplementary Table Legends**

**Supplementary Table 1**. Viruses predicted to use alternate translation tables. This table includes the ID (UHGV) or Accession (INPHARED) for sequences predicted to use alternate translation tables, as well as predicted translation table and viral taxonomy.

**Supplementary Table 2**. Annotation statistics. This table includes summary statistics relating to the annotations produced in these analyses.
